# Chromosome Instability and Mosaic Aneuploidy in Neurodegenerative and Neurodevelopmental Disorders

**DOI:** 10.3389/fgene.2019.01092

**Published:** 2019-11-07

**Authors:** Huntington Potter, Heidi J. Chial, Julbert Caneus, Mihret Elos, Nina Elder, Sergiy Borysov, Antoneta Granic

**Affiliations:** ^1^Department of Neurology, Rocky Mountain Alzheimer’s Disease Center, University of Colorado, Aurora, CO, United States; ^2^Linda Crnic Institute for Down Syndrome, University of Colorado, Aurora, CO, United States; ^3^NanoScience Technology Center, University of Central Florida, Orlando, FL, United States; ^4^Department of Math and Science, Saint Leo University, Saint Leo, FL, United States; ^5^AGE Research Group, Institute of Neuroscience, Newcastle University, Newcastle upon Tyne, United Kingdom; ^6 ^Newcastle University Institute for Ageing, NIHR Newcastle Biomedical Research Centre, Newcastle upon Tyne, United Kingdom; ^7^Newcastle upon Tyne Hospitals, NHS Foundation Trust, Newcastle upon Tyne, United Kingdom

**Keywords:** Alzheimer’s disease, Mosaic aneuploidy, Frontotemporal lobar degeneration, Neuronal apoptosis, Huntington’s disease (HD)

## Abstract

Evidence from multiple laboratories has accumulated to show that mosaic neuronal aneuploidy and consequent apoptosis characterizes and may underlie neuronal loss in many neurodegenerative diseases, particularly Alzheimer’s disease and frontotemporal dementia. Furthermore, several neurodevelopmental disorders, including Seckel syndrome, ataxia telangiectasia, Nijmegen breakage syndrome, Niemann–Pick type C, and Down syndrome, have been shown to also exhibit mosaic aneuploidy in neurons in the brain and in other cells throughout the body. Together, these results indicate that both neurodegenerative and neurodevelopmental disorders with apparently different pathogenic causes share a cell cycle defect that leads to mosaic aneuploidy in many cell types. When such mosaic aneuploidy arises in neurons in the brain, it promotes apoptosis and may at least partly underlie the cognitive deficits that characterize the neurological symptoms of these disorders. These findings have implications for both diagnosis and treatment/prevention.

## Introduction

Age-associated neurodegenerative diseases exhibit different brain pathologies and different clinical features, and all are associated with reduced neuronal numbers in specific brain regions. Furthermore, when caused by a mutation, each disorder evidently involves a unique pathogenic pathway because the mutant proteins are usually involved in very different physiological processes. Indeed, the normal function of the associated mutant gene should provide insights into that specific disease’s pathogenic pathway. For example, the mutations that cause autosomal dominant familial Alzheimer’s disease (FAD) arise in only three genes: the amyloid precursor protein (*APP*) gene, the presenilin 1 (*PSEN1*) gene, and the presenilin 2 (*PSEN2*) gene, all three of which encode proteins involved in the production of the Aβ peptide, which is the main pathogenic molecule of AD (Goate and Hardy, 2012; Hardy, 2017). Specifically, PSEN1 and PSEN2 are components of the γ-secretase complex, which, together with the β-secretase enzyme, cleaves APP to release Aβ. In contrast, patients with frontotemporal dementia (also called frontotemporal lobar degeneration, FTLD) exhibit clinical and pathological characteristics that differ from AD, and most of the autosomal dominantly inherited familial forms of FTLD are caused by a mutation in the *MAPT/Tau* gene, by a mutation in the progranulin (*PRGN*) gene, or by a triplet repeat expansion in the *C9ORF72* gene, all three of which carry out vastly different normal functions in the cell and are also unrelated to the genes mutated in FAD (Rademakers et al., 2012). Interestingly, in some families, instead of or in addition to FTLD, the triplet repeat expansion in the *C9ORF72* gene can cause familial amyotrophic lateral sclerosis, a motor neuron degenerative disease that is associated with cognitive decline only during later stages of the disease. Huntington’s disease similarly exhibits a unique pathology and clinical course and is caused by a triplet repeat expansion in the huntingtin (*HTT*) gene, whose normal function is also apparently different from any of the genes associated with familial forms of AD or FTLD (Podvin et al., 2019). Thus, there appears to be no common feature of neurodegenerative disorders beyond the fact that they all result in neuronal loss.

Neurodevelopmental disorders are associated with different pathologies and clinical phenotypes, although they again usually include microcephaly or another indication of a defect in neurogenesis or neuronal survival. For example, ataxia telangiectasia and the related disorder Seckel syndrome are caused by mutations in the *ATM* and *ATR* genes, respectively, which encode two related protein kinases. Because both ataxia telangiectasia and Seckel syndrome appear to involve the loss of neurons, they might be considered neurodegenerative disorders without an essential aging component associated with their underlying mechanisms. Down syndrome also results in reduced neuronal numbers in key brain regions during development, and, interestingly, every person with Down syndrome develops AD brain neuropathology by age 30–40, which usually leads to dementia by age 50–60 (Hartley et al., 2015; Hithersay et al., 2019).

Because these multiple disorders have different pathologies and different clinical symptoms and they involve different pathogenic pathways, as evidenced by the different genes in which causal mutations can arise, it is reasonable to hypothesize that each disorder is distinct and would require different approaches to therapy and prevention. However, if it were possible to identify a key step that is shared among the pathogenic pathways of many neurodegenerative and/or neurodevelopmental disorders, then it would also be reasonable to hypothesize that insights into the causal mechanism might be gained and the potential for a common approach to the development of new therapies might be recognized. Of course, any shared mechanistic features that are identified must also be considered as potentially a mere correlate of the degenerative process rather than as an *essential* step in the pathogenic pathways. To distinguish between these two hypotheses, the strength of the genetics can be exploited because it is self-evident that a direct effect of a mutant gene is likely to be part of the causal mechanism. If multiple neurodevelopmental and neurodegenerative disease-causing mutations impact a common pathogenic step, then that step should be considered a potential key to preventing the neurodegeneration and should thus serve as a prime target for the development of therapeutic interventions that could be applied to multiple disorders.

Over the past decade, we and others have identified a potential common step in the pathogenic pathways that lead to multiple distinct neurodegenerative and neurodevelopmental disorders. Specifically, mutations in genes linked to many of these disorders have been shown to lead to chromosome segregation defects and mosaic aneuploidy in cell types throughout the body, including in brain neurons, which likely contributes to the neuronal cell loss/apoptosis that underlies their neurological features.

### Mosaic Aneuploidy in Alzheimer’s Disease

Mosaic aneuploidy/hyperploidy, including trisomy 21, was first hypothesized (Potter, 1991) and has been most thoroughly investigated in AD (Geller and Potter, 1999; Yang et al., 2001; Kingsbury et al., 2006; Mosch et al., 2007; Thomas and Fenech, 2008; Iourov et al., 2009b; Arendt et al., 2010; Iourov et al., 2011). Arendt and colleagues’ extensive study of brains from AD patients showed that 20–30% of brain neurons are aneuploid during the early preclinical stages of AD and that their specific loss in later stages of the disease can account for 90% of the neuronal atrophy observed at autopsy (Arendt et al., 2010). Somatic mosaic aneuploidy can also be detected in cells from peripheral tissues of AD patients, including fibroblasts, peripheral blood mononuclear cells, and buccal cells (Potter et al., 1995; Migliore et al., 1997; Geller and Potter, 1999; Migliore et al., 1999; Trippi et al., 2001; Thomas and Fenech, 2008), providing an opportunity for early detection.

The specific finding that trisomy 21 mosaicism occurs in many tissues in AD patients, including in the brain, is highly relevant because people with Down syndrome are usually fully trisomic for chromosome 21 due to chromosome mis-segregation during meiosis, every person with Down syndrome develops AD brain neuropathology by 35 years of age, the majority of people with Down syndrome develop AD dementia by age 60, and nearly all people with Down syndrome who die after age 35 have dementia (Glenner and Wong, 1984; Wisniewski et al., 1985; Epstein, 1990; Hartley et al., 2015; Hithersay et al., 2019). The *APP* gene is located on chromosome 21, and its presence in three copies in Down syndrome is presumed to underlie the development of early-onset AD in this population (Hartley et al., 2015). Support for this conclusion comes from the observation that individuals who carry an FAD mutation in the *APP* gene or who have three copies of the *APP* gene due to a local duplication on one chromosome (Rovelet-Lecrux et al., 2006; Sleegers et al., 2006) will develop autosomal-dominant early-onset AD. Furthermore, individuals who only have 1–10% trisomy 21 cells and show no features of Down syndrome also develop early-onset, sporadic AD dementia, suggesting that even low levels of trisomy 21 mosaicism can lead to AD (reviewed in Potter, 1991). Evidence that three copies of the *APP* gene are not only *sufficient* but also *necessary* to cause AD is provided by the fact that rare individuals who have full trisomy 21 and all of the clinical and physiological features of Down syndrome but have only two copies of the *APP* gene due to a localized deletion on chromosome 21 fail to develop AD symptoms or AD pathology even at an old age (Prasher et al., 1998; Doran et al., 2017).

Evidence that an underlying susceptibility to chromosome segregation defects may be associated with an increased risk of AD was first provided by a study showing that women who were 35 or younger when they gave birth to a child with Down syndrome have a fivefold increased risk of developing AD later in life compared to control mothers or compared to mothers who gave birth to a child with Down syndrome after age 35 (Schupf et al., 1994). More direct support for the idea that both trisomy 21 and AD are associated with an underlying predisposition for chromosome mis-segregation comes from a study of cultured peripheral blood lymphocytes from mothers under the age of 35 who gave birth to a child with Down syndrome (Migliore et al., 2006). In that study, they used fluorescence in situ hybridization probes for chromosomes 21 and 13 and observed significantly higher levels of chromosome non-disjunction involving both chromosomes within the first cell cycle in the lymphocytes from mothers who gave birth to a child with Down syndrome compared to control mothers who had not had a miscarriage and whose children did not have genetic disorders (Migliore et al., 2006). Taken together, these findings suggest that an underlying predisposition for chromosome instability may lead to increased AD risk.

The association of chromosome instability and aneuploidy with AD has been reinforced by mechanistic studies. The key proteins whose mutant genes cause the majority of FAD—the presenilin proteins (PSEN1 and PSEN2) and the amyloid precursor protein (APP)—localize to centromeres/kinetochores, centrosomes, and/or the nuclear envelope (Li et al., 1997; Annaert et al., 1999; Honda et al., 2000; Jeong et al., 2000; Kimura et al., 2001; Nizzari et al., 2007a; Nizzari et al., 2007b; Young-Pearse et al., 2010; Judge et al., 2011). Furthermore, FAD mutations in *PSEN1* or *APP* cause mitotic spindle abnormalities and aneuploidy in transgenic mice and in transfected cells (Boeras et al., 2008; Granic et al., 2010). Treatment of karyotypically normal cells with oligomeric Aβ peptide, which is the product of PSEN1- and PSEN2-dependent cleavage of APP, also disrupts the mitotic spindle and induces chromosome mis-segregation and aneuploidy by competitively inhibiting certain microtubule motors, particularly Kinesin-5/KIF11/Eg5, MCAK/KIF2C, and KIF4A, in both cell culture experiments and in *Xenopus* egg extracts (Boeras et al., 2008; Borysov et al., 2011). These mechanistic studies established that cell cycle defects and the resultant mosaic aneuploidy are a direct effect of FAD mutant genes and are thus likely to be part of the AD pathogenic pathway and not merely a correlate of neurodegeneration in the brain.

The role of chromosome aneuploidy in AD suggests that studying mechanisms that regulate mitosis may lead to novel insights into AD. For example, Shugoshin-1 (SGO1) encodes a protein that is involved in chromosome cohesion and is needed for normal chromosome segregation, and SGO1 haploinsufficiency leads to chromosome missegregation and tumorigenesis (Yamada et al., 2012). Building on the role of aneuploidy in AD, Rao and Yamada and colleagues hypothesized that *SGO1* heterozygous knockout mice may serve as a potential model of sporadic late-onset AD, and they indeed discovered some AD-related pathology as the mice aged, which was associated with prolonged mitosis and spindle checkpoint activation (Rao et al., 2018a; Rao et al., 2018b).

### Mosaic Aneuploidy in Frontotemporal Lobar Degeneration

In addition to AD, mosaic aneuploidy has been observed in FTLD (Rossi et al., 2013; Rossi et al., 2014; Caneus et al., 2018). In earlier studies, mosaic aneuploidy was reported in skin fibroblasts and peripheral blood lymphocytes from FTLD patients and in splenic lymphocytes from mouse models of FTLD-MAPT (Rossi et al., 2008; Rossi et al., 2013; Rossi et al., 2014). In the subsequent study, we found mosaic aneuploidy and associated apoptosis in both neuronal and non-neuronal brain cells from patients with familial FTLD who carry a mutation in the *MAPT/Tau* gene (Caneus et al., 2018). Expression of FTLD-causing mutant MAPT induced mitotic spindle abnormalities, chromosome mis-segregation, aneuploidy, and apoptosis in neurons and other cells in the brains of transgenic mice and in transfected cells (Caneus et al., 2018). Furthermore, we showed in our FTLD study that apoptosis occurs in the same brain neurons that are aneuploid and that, in cultured cells expressing FTLD-causing mutant forms of human MAPT, apoptosis follows and depends upon aneuploidy-generating cell cycle defects (Caneus et al., 2018). If the cell cycle is blocked by inhibiting the interaction between MDM2 and p53 by treatment with low doses of Nutlin-3 at 24 h after expression of FTLD-causing mutant MAPT, no aneuploid cells arise (Caneus et al., 2018). Importantly, treatment with Nutlin-3 also blocks apoptosis, indicating that the toxicity of mutant MAPT depends on cells aberrantly proceeding through the cell cycle and becoming aneuploid.

Evidence linking neuronal aneuploidy, neurodegeneration, and MAPT was reported recently by two other groups in *Drosophila* models of FTLD. Specifically, a study by Bougé and Parmentier showed that excess Tau causes mitotic spindle defects, aneuploidy, and apoptosis in neurons by inhibiting the microtubule-dependent motor protein Kinesin-5 (Bouge and Parmentier, 2016). Similar results have been reported by Malmanche et al. who examined photoreceptors and brain neurons in *Drosophila* and found that adult-onset neurodegeneration mediated by MAPT overexpression included the generation of aneuploid cells (Malmanche et al., 2017). The former result is of particular interest in view of our previous finding that Aβ induces chromosome mis-segregation and aneuploidy by competitively inhibiting the activity of Kinesin-5/KIF11/Eg5 (Borysov et al., 2011). Thus, causal mutations leading to AD and FTLD-MAPT appear to lead to chromosome mis-segregation, aneuploidy, and apoptosis through inhibition of the same target enzyme: Kinesin-5/KIF11/Eg5.

In addition to MAPT-FTLD, we have found that mosaic neuronal aneuploidy and dependent apoptosis are also characteristic of brains of individuals with sporadic FTLD or with FTLD caused by mutations in *PRGN* or by triplet repeat expansions in *C9ORF72* (Elos and Caneus et al., unpublished results, manuscript in preparation).

It is likely that other neurodegenerative diseases are also associated with mosaic aneuploidy in the brain. For example, autism spectrum disorder (Yurov et al., 2007; Iourov et al., 2008), ataxia telangiectasia (McConnell et al., 2004; Iourov et al., 2009a; Iourov et al., 2009b), and Lewy body disease, which includes Parkinson’s disease (Yang et al., 2015), have all been reported to exhibit either general hyperploidy or mosaic aneuploidy for numerous chromosomes in brain and/or peripheral tissues. Our laboratory also has preliminary evidence for mosaic aneuploidy in both brain cells and fibroblasts from Huntington’s disease patients (Elos and Caneus et al., unpublished results, manuscript in preparation).

### Mosaic Aneuploidy in Neurodevelopmental Disorders

Mosaic aneuploidy in neurons and other types of cells also characterizes neurodevelopmental disorders. For example, loss-of-function mutations in the ataxia telangiectasia mutated and Rad3-related (ATR) encoded kinase cause Seckel syndrome, a rare autosomal recessive disorder characterized by pre- and postnatal growth delays, microcephaly, and intellectual disability. Loss of ATR function and of the related kinase ataxia telangiectasia mutated (ATM) have been linked to defective DNA repair, which has been assumed to cause the genomic instability, including aneuploidy, observed in these disorders and to make ataxia telangiectasia patients prone to cancer (Wright et al., 1998; Spring et al., 2002; Shen et al., 2005; Murga et al., 2009; Lang et al., 2016; Yazinski and Zou, 2016; Blackford and Jackson, 2017; Quek et al., 2017). Previous studies showed that ATR localizes to centrosomes (Zhang et al., 2007) and that loss of ATR function causes centrosome overduplication (Alderton et al., 2004; Collis et al., 2008; Stiff et al., 2016) and genomic instability (Casper et al., 2004; Mokrani-Benhelli et al., 2013). In a recent study, Kabeche and colleagues reported a mechanism by which loss of ATR function leads to chromosome mis-segregation and aneuploidy (Kabeche et al., 2018; Saldivar and Cimprich, 2018). Specifically, they elegantly demonstrated that ATR localizes to centromeres and is required for proper chromosome segregation, in addition to and *independent of* its roles in DNA damage repair and replication stress responses (Kabeche et al., 2018). Although not discussed by Kabeche and colleagues or in previous publications, the links between ATR and mitosis provide an explanation for how reduced ATR function and subsequent aneuploidy may underlie the neuronal cell loss during development that leads to microcephaly and cognitive dysfunction, the major clinical, pathological, and disabling features of Seckel syndrome: reduced ATR function results in aneuploidy that leads to neuronal apoptosis.

In addition to Seckel syndrome, mosaic aneuploidy has been observed in brain neurons in ataxia telangiectasia itself (Iourov et al., 2007; Iourov et al., 2009a; Iourov et al., 2009b) and in Niemann–Pick type C disease (Granic and Potter, 2013), and in peripheral cells in Nijmegen breakage syndrome (Vessey et al., 1999; Shimada et al., 2009; Shimada et al., 2010; Hou et al., 2012), Fanconi anemia (Nalepa et al., 2013), and xeroderma pigmentosum (Amiel et al., 2004). All of these developmental disorders are characterized by microcephaly or other evidence of poor neurogenesis and/or of neuronal loss, and all are associated with cognitive disfunction.

### Mechanisms by Which Neuronal Aneuploidy and Apoptosis Can Arise

Because neurons have been traditionally considered to be post-mitotic (Bhardwaj et al., 2006), it has been unclear how extensive mosaic aneuploidy can arise in neurodegenerative or neurodevelopmental disorders. More recently, it has become appreciated that neurogenesis is more widespread than previously thought and that the capacity for neurogenesis continues into old age, even if not normally utilized (Zhao et al., 2008; Spalding et al., 2013; Boldrini et al., 2018; Sorrells et al., 2018). In the adult brain, three processes have been identified that may generate the neuronal aneuploidy observed at autopsy in patients with AD, FTLD-MAPT, and other neurodegenerative and neurodevelopmental disorders. In principle, the generation and accumulation of aneuploidy in dividing or regenerating cell populations might arise by both genetic and environmental stressors at any time in life (discussed in Potter, 1991; Oromendia and Amon, 2014). Indeed, there is strong evidence that neurogenesis can occur throughout life in several regions of the brain (Zhao et al., 2008; Mu and Gage, 2011; Spalding et al., 2013; Ernst et al., 2014). Furthermore, data from many studies provide evidence that neurogenesis can be induced in many brain regions in adult mice and rats in response to brain damage and attempted self-repair by the brain (Zhou et al., 2004; Zhao et al., 2008; Spalding et al., 2013; Zheng et al., 2013; Ibrahim et al., 2016), or as part of an ongoing process in the sub-ventricular/granular zone of the brain (Eriksson et al., 1998; Hallbergson et al., 2003; Sakamoto et al., 2014). Thus, neuronal damage and the mitotic defects evident in AD, FTLD-MAPT, and other neurodegenerative and neurodevelopmental disorders could result in the production of new aneuploid neurons, which would not be fully functional and would be particularly prone to apoptosis and degeneration. Indeed, aneuploidy has been shown to promote cell death, including neurodegeneration, in many experimental systems (Rajendran et al., 2008; Kai et al., 2009; Arendt et al., 2010; Oromendia and Amon, 2014).

The second potential mechanism for the generation of neuronal aneuploidy in neurodegenerative disease is cell cycle reentry. Neurons in the AD brain express phospho-proteins usually detected only during mitosis, such as cyclin B1, cyclin D1, cdc2, and Ki67 (Vincent et al., 1996; McShea et al., 1997; Vincent et al., 1997; Yang et al., 2001; Arendt, 2012). In AD mice, the loss of preexisting neurons induces the remaining neurons to reenter the cell cycle (Lopes et al., 2009). Indeed, Aβ has been shown to induce the expression of mitotic proteins and cell cycle reentry in mature neurons in culture (Majd et al., 2008; Absalon et al., 2013; Seward et al., 2013), which we have confirmed (Nina Elder, unpublished observation).

The third potential mechanism for generating aneuploid neurons is based on the recent discovery that striatal astrocytes can transdifferentiate into new neurons capable of forming functional neuronal circuits with preexisting neurons following ischemic brain injury (Magnusson et al., 2014; Duan et al., 2015). This finding suggests that at least some of the aneuploid neurons in AD and FTLD-MAPT brains may be derived from the aneuploid glia that we have shown are present in our preliminary and published studies. In additional preliminary studies, we have found that low numbers of primary astrocytes exposed to Aβ in culture can begin to express the neuronal marker NeuN (Nina Elder, unpublished observation). Taken together, these findings provide evidence that aneuploidy can arise *de novo* in mature neurons by cell cycle reactivation or can be carried over from previously dividing cells that generate new neurons. It is reasonable that age may exacerbate all of these processes because neuronal and non-neuronal aneuploidy have been shown to increase with age (Arendt et al., 2009; Yurov et al., 2009; Yurov et al., 2010; Fischer et al., 2012; Fantin et al., 2019). Aging is also associated with increasing total exposure to environmental stressors, some of which can promote chromosome missegregation and aneuploidy (for reviews, see Potter, 1991; Iourov et al., 2013).

In addition to the close and mechanistic association between aneuploidy and induced apoptosis discussed above, multiple reports in different systems have shown that aneuploid or other copy number variant cells are prone to degeneration/apoptosis (Oromendia and Amon, 2014; Ohashi et al., 2015; Potter et al., 2016; Andriani et al., 2017; Chronister et al., 2019). As mentioned earlier, Arendt and colleagues conducted a pathological study of AD patients’ brains across the disease spectrum and showed that neuronal aneuploidy arises before neurodegeneration or clinical symptoms are evident (Arendt et al., 2010). Specifically, they found that the number of aneuploid neurons increases steadily from around 10% in normal controls to around 30% during the early preclinical stages of AD and then declines back to around 10% during the transition from preclinical AD to severe AD when neuronal loss occurs. In addition, they calculated that the loss of aneuploid, but not diploid, neurons accounted for 90% of the neuronal atrophy observed at autopsy of late-stage AD brains (Arendt et al., 2010). Based on their findings, it can be concluded that: 1) aneuploidy in neurons arises in the AD brain before extensive neuronal cell loss occurs and thus the aneuploidy is not likely to be caused by neurodegeneration/neuronal apoptosis, and 2) the vast majority of later neuronal cell loss selectively affects aneuploid neurons, indicating that the neurodegeneration is likely caused by a cell-autonomous cell cycle defect in the neurons themselves rather than by a tissue-wide mechanism (such as an unidentified, diffusible toxic insult released from damaged cells). Possible cell-autonomous effects of aneuploidy that could contribute to cell death include DNA replication stress (Yurov et al., 2011) and proteotoxic stress (Oromendia et al., 2012).

### Linking Development and Aging: A Role for Catalysts in Age-Associated Proteinopathies

In view of these considerations, we note that developmental disorders, such as Seckel syndrome, ataxia telangiectasia, Niemann–Pick type C, Nijmegen breakage syndrome, Fanconi anemia, and xeroderma pigmentosum, all of which lead to neuronal apoptosis, degeneration, and microcephaly, result from mutations in genes whose products impact mitosis, directly or indirectly. In contrast, aging-associated neurodegenerative diseases, such as AD, FTLD, Lewy body disease (Yang et al., 2015), and potentially Huntington’s disease (Sathasivam et al., 2001; Elos and Caneus et al., unpublished results) and prion disease (Basu et al., 1998; Borchsenius et al., 2000; Nieznanska et al., 2012) all develop abnormal protein deposits in the brain in addition to aneuploidy. The formation of these deposits apparently involves not only the seminal protein itself but often requires inflammation or other aging-associated catalysts. For example, inheritance of the ε4 allele of the apolipoprotein E (*APOE*) gene is the strongest risk factor for the development of AD besides age itself. Interestingly, the *APOE4* allele and an AD-linked *PSEN1* polymorphism have each been shown to increase the risk of meiosis II chromosome segregation errors, leading to Down syndrome, and a mother carrying both the *APOE4* allele and the *PSEN1* polymorphism has an even higher risk of a trisomy 21 conception (Avramopoulos et al., 1996; Petersen et al., 2000; Rodriguez-Manotas et al., 2007; Bhaumik et al., 2017). Indeed, a recent study of older adults with Down syndrome reported that those who were *APOE4* carriers were at increased risk of both dementia and death (Hithersay et al., 2019). Notably, ApoE, particularly ApoE4, catalyzes the conversion of Aβ into the toxic oligomers that directly disrupt the mitotic spindle and chromosome segregation and also leads to amyloid deposition (Potter and Wisniewski, 2012). A similar co-pathological protein likely exists in prion disease too, although this exacerbating protein has been shown not to be ApoE (Tatzelt et al., 1996). This two-hit mechanism involving a mutant aggregation-prone protein plus an amyloid catalyst may underlie the fact that, in AD, amyloid deposits, symptoms, and aneuploidy all arise with aging. Similar two-hit mechanisms may underlie other aging-associated neurodegenerative diseases and neurodevelopmental disorders. Furthermore, the region-specific expression of the second hit (such as with ApoE in AD) may underlie the region-specific pathology and neuronal loss in different disorders.

### Constitutional Aneuploidy in the Normal Brain

In addition to its association with neurodegenerative and neurodevelopmental disorders, aneuploidy and possibly copy number variations on a smaller scale are considered potential contributors to diversity in brain function (Iourov et al., 2009b; Mkrtchyan et al., 2010; Bushman and Chun, 2013; Rohrback et al., 2018). Although extensive whole chromosome aneuploidy has not been found by all investigators (Knouse et al., 2014), it is likely that new methods will reveal more aneuploid cells in both normal aged and diseased brains (Caneus et al., 2017).

### Summary

**Figure 1:**
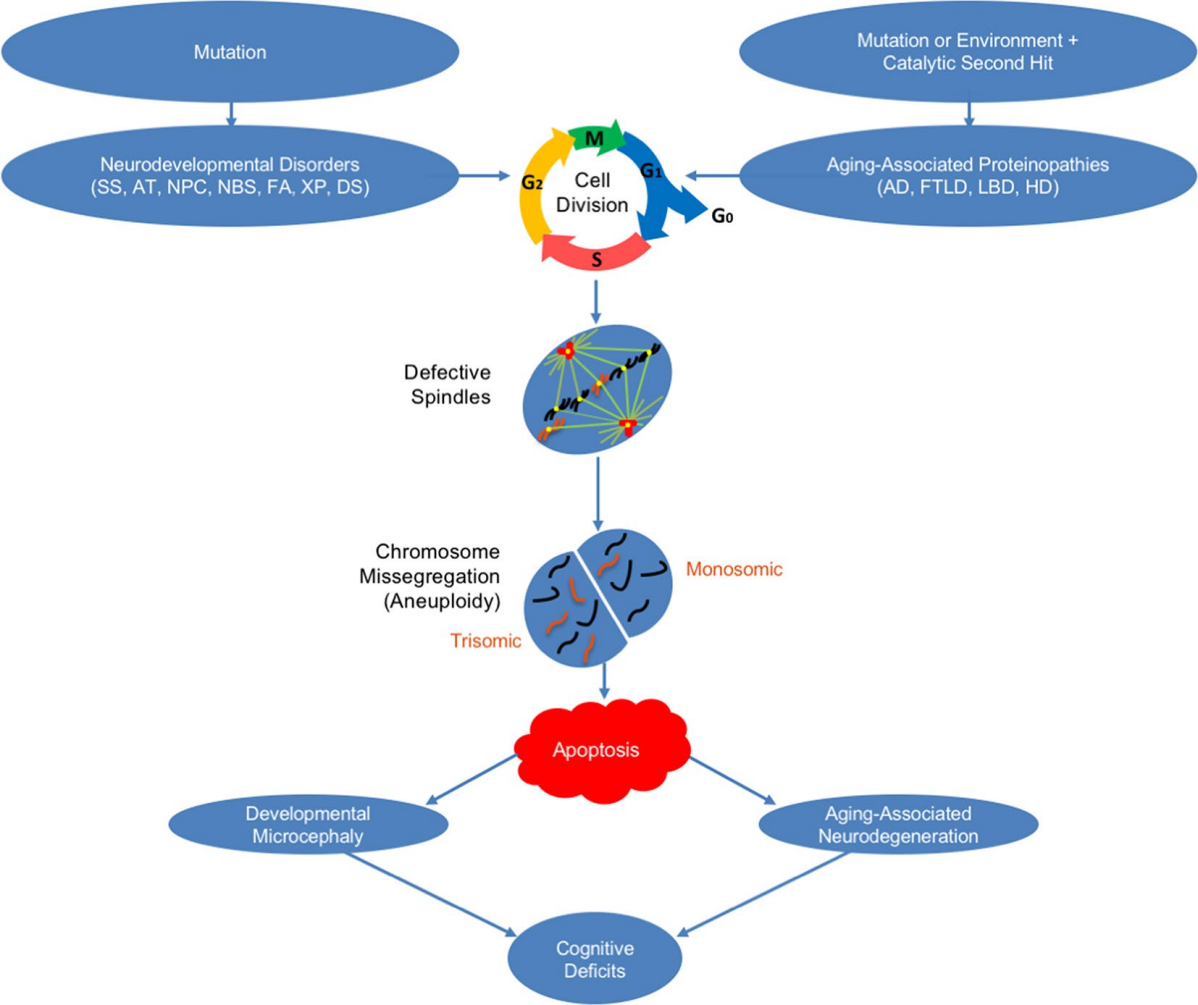
How mosaic aneuploidy may lead to neuronal loss in neurodegenerative and neurodevelopmental disorders. Shown is a schematic of how mosaic aneuploidy may occur in neurons leading to apoptosis and cognitive deficits in neurodevelopmental and aging-associated neurodegenerative disorders. Seckel syndrome (*SS*), ataxia telangiectasia (*AT*), Niemann–Pick type C disease (*NPC*), Nijmegen breakage syndrome (*NBS*), Fanconi anemia (*FA*), xeroderma pigmentosum (*XP*), Down syndrome (*DS*), Alzheimer’s disease (*AD*), frontotemporal lobar degeneration (*FTLD*), Lewy body disease (*LBD*), and Huntington’s disease (*HD*).

In sum, recent work reinforces our emerging understanding of the important role that chromosome mis-segregation and mosaic aneuploidy in neurons may play in an ever-growing list of both neurodevelopmental disorders and aging-associated neurodegenerative disorders (Figure 1). These findings have potentially important implications for the development of: 1) novel diagnoses because, as discussed, in addition to neurons in the brain, peripheral cells also exhibit mosaic aneuploidy in these disorders, and 2) innovative preventions/treatments because interventions can now be sought that specifically promote correct chromosome segregation in the presence of aneugenic mutations and/or aneugenic protein structures that lead to neuropathogenesis.

## Data Availability Statement

The datasets generated for this study are available on request to the corresponding author.

## Author Contributions

All authors contributed to the design and/or execution of the experiments. HP and HC wrote the manuscript. JC primarily designed the figure.

## Conflict of Interest

The authors declare that the research was conducted in the absence of any commercial or financial relationships that could be construed as a potential conflict of interest.
